# Current State and Future of Artificial Intelligence in Pediatric Interventional Radiology: A Narrative Review

**DOI:** 10.3390/diagnostics16121918

**Published:** 2026-06-20

**Authors:** Abdulaziz Mohammad Al-Sharydah

**Affiliations:** Diagnostic and Interventional Radiology Department, Imam Abdulrahman Bin Faisal University, King Fahd Hospital of the University, Khobar City P.O. Box 31952, Saudi Arabia; amsharydah@iau.edu.sa; Fax: +966-013-8676697

**Keywords:** artificial intelligence, clinical translation, interventional radiology, pediatrics, robotics, vascular

## Abstract

Artificial intelligence (AI) is reshaping the field of diagnostic radiology; however, its applications in interventional radiology and pediatric interventional radiology (PIR) remain limited despite clear clinical needs and the rich multimodal data environment characteristic of pediatric procedural care. In this narrative review, I summarize the current state of AI technologies relevant to PIR and outline future perspectives for their clinical integration. Peer-reviewed literature and position statements identified through MEDLINE/PubMed, Embase, Scopus, and major society publications up to the first quarter of 2026 are synthesized, focusing on AI applications across the PIR care pathway, including dose-sparing image acquisition and reconstruction, automated image interpretation and computer-aided diagnosis, data-driven procedural planning and navigation, and post-procedural risk prediction and monitoring. After briefly introducing core machine learning and deep learning concepts, pediatric-specific challenges are discussed, including radiation sensitivity, growth-related anatomical variability, regulatory constraints, and the scarcity of large, annotated datasets, as well as existing and emerging applications along the PIR care pathway: AI-assisted dose reduction and image reconstruction, automated image interpretation, segmentation, and computer-aided diagnosis; data-driven procedural planning, including three-dimensional modelling, augmented reality, AI-enabled/AI-adjacent robotics, and AI-directed procedural navigation; and post-procedural risk prediction and outcome monitoring. Finally, emerging paradigms, including explainable AI, federated learning, and multimodal integration, are highlighted, and research priorities, collaborative frameworks, and governance principles required to ensure safe, equitable, and effective AI deployment in PIR are outlined. In doing so, this review delineates the current evidence gaps and priority directions for clinically meaningful AI adoption in PIR. Although AI has the potential to improve patient care, it has not yet been specifically designed, validated, or deployed in children. Existing work demonstrates feasibility across the PIR workflow, but most tools remain weakly linked to pediatric clinical endpoints.

## 1. Introduction

Artificial intelligence (AI), including machine learning (ML) and deep learning (DL), encompasses computational methods that learn complex mappings from high-dimensional data, such as images, signals, clinical variables, and text [1,2,3]. In medical imaging, AI has progressed rapidly in diagnostic radiology, especially in high-volume adult imaging, whereas interventional radiology (IR), particularly pediatric IR (PIR), has seen limited, fragmented use, consistent with the findings of prior reports describing only a small number of early, exploratory PIR AI applications [1,2,3]. Most angiography suites rely only on manufacturer-embedded processing [1,2,3]. Pediatric subspecialties, such as cardiology, congenital heart disease, liver transplantation, and perinatology, generate rich multimodal data, yet remain underserved by AI for decision support, risk prediction, and workflow optimization [4,5,6]. Compared to outcome-prediction models in these fields, PIR lacks equivalent AI-based bedside support [3,5,6]. In IR and PIR, AI has been explored for workflow optimization, intraprocedural imaging, dose management, wire and catheter navigation, and postprocedural outcome prediction. Most of these implementations remain at an early or investigational stage. In this context, AI-enabled robotics describes platforms that incorporate AI-derived image analysis or decision-support modules, while AI-adjacent robotics refers to systems that generate rich procedural data streams but whose control logic remains predominantly rule-based and operator-directed [3,7,8,9]. In children, these tools intersect with the following priorities: minimizing radiation, accommodating small, evolving anatomy, and integrating longitudinal data across growth and development [10,11,12].

The American College of Radiology (ACR) Pediatric AI Workgroup describes a “pediatric AI gap,” reporting that among >200 cleared imaging AI tools, only a few explicitly target pediatrics, and none primarily address pediatric computer-aided detection, triage, or diagnosis [10]. In general, most pediatric AI tools are trained on adult data, despite major biological and developmental differences, raising equity and safety concerns when applied to children [10,13,14].

In this narrative review, AI and DL methods relevant to PIR are synthesized and PIR-specific challenges, ethical and regulatory issues, emerging technologies, and priority research directions are outlined. This paper reflects the perspective of clinicians and researchers with active roles in PIR and AI-enabled procedural platforms [8,15].

### Search Strategy and Selection

Consistent with published guidance on the structured reporting of narrative reviews, this work was designed as a narrative, clinically oriented review rather than a formal systematic review, given the heterogeneity and early, largely feasibility-level nature of PIR–related AI studies. Narrative searches of MEDLINE/PubMed, Embase, and Scopus were performed up to the first quarter of 2026 using combinations of terms such as “pediatric interventional radiology,” “pediatric cardiac catheterization,” “artificial intelligence,” “machine learning,” “deep learning,” “robotic,” “navigation,” “dose reduction,” and “risk prediction,” and were supplemented by key guidelines, position statements, and white papers from professional societies including the ACR, the Society of Interventional Radiology (SIR), the Society for Pediatric Radiology (SPR), and the Cardiovascular and Interventional Radiological Society of Europe (CIRSE), as well as related pediatric subspecialty groups in cardiology, transplantation, and neuro-oncology. English-language, peer-reviewed articles and conference papers describing AI or robotic methods with direct or conceptual relevance to PIR workflows in pediatric or mixed-age populations were prioritized, whereas purely adult diagnostic studies without interventional implications, non-peer-reviewed material, and non-medical technical reports were generally excluded.

Candidate articles were screened by title and abstract, followed by full-text review where appropriate. When overlapping publications were identified, the most recent, influential (higher-impact), or more comprehensive was preferentially cited. This process yielded approximately 70–80 primary and review articles, along with several society statements, which were synthesized into a single clinically oriented framework spanning diagnostic, interventional, technical, and policy perspectives. Accordingly, this review should be interpreted as a hypothesis-generating narrative overview rather than a systematic evidence inventory. Given the heterogeneity, small sample sizes, and largely feasibility-level nature of current PIR-related AI reports, this work is presented as a structured, hypothesis-generating narrative review with qualitative appraisal of available technologies, rather than as a formal technology assessment with flowcharts or comparative effectiveness algorithms.

## 2. Current State of AI in PIR

### 2.1. Overview of Existing AI Technologies

Imaging AI commonly uses supervised learning (e.g., logistic regression, random forests, and support vector machines), unsupervised learning (e.g., clustering and dimensionality reduction), and DL architectures, such as convolutional neural networks (CNNs), recurrent networks, and transformers [1,2]. CNNs have achieved near-expert performance in selected cardiology tasks, including arrhythmia classification from single-lead ECGs, automated echocardiographic view recognition and segmentation, and right ventricular motion analysis on cardiac MRI, suggesting the applicability of DL to temporal and volumetric data encountered in interventional imaging [1,2,16].

Desai et al. highlighted PIR AI opportunities in scheduling, fluoroscopic dose reduction, outcome prediction, equipment selection, robotics, human–computer interaction, and education [3]. AI denoising algorithms have been applied for real-time fluoroscopy, acquisition, and digital subtraction angiography (DSA), and big-data-driven exposure control that automatically optimizes the kV, mA, filtration, pulse width, focal spot, detector dose, and collimation as the C-arm position or angulation changes [3,17,18]. In 2025, Siemens Healthineers introduced OptiqAI for Artis interventional platforms (genio/icono/pheno), an AI-powered imaging chain that combines real-time image denoising with big-data–driven automatic exposure control across fluoroscopy, acquisition, and digital subtraction angiography, dynamically adjusting tube voltage, tube current, copper prefiltration, focal spot size, pulse width, detector dose, and collimation to maintain the requested image quality at the lowest procedure time and reasonable dose) [17].

In the image-guiding portfolio of GE Healthcare, the Allia interventional platforms incorporate the AutoRight intelligent image chain, which uses artificial intelligence to optimize multiple imaging and dose parameters in real time during fluoroscopy and acquisition, aiming to maintain consistent image quality at the lowest reasonable dose across a broad range of procedures [18].

ML models combining clinical, laboratory, demographic, and imaging data can predict responses to intra-arterial tumor therapies, classifying responders and non-responders in small cohorts [9]. These histology-agnostic, multimodal concepts are transferable to pediatric tumor ablation, venous interventions, and embolization of vascular malformations, where current scoring systems are rudimentary [3,5,9].

As of early 2026, no AI software as a medical device product has a labeled intended use specific to PIR; pediatric services rely on adult or mixed population systems without pediatric-specific validation. Similarly, the ACR Pediatric AI white paper notes that among >200 Food and Drug Administration (FDA) approved imaging AI products, only a few indicate pediatric use, and none target computer-aided diagnosis (CAD)/triage/diagnosis in interventional workflows [10]. For congenital heart disease, Holt et al. reviewed AI across echocardiography, computed tomography (CT), magnetic resonance imaging (MRI), and catheterization, including DL-based segmentation, outcome prediction, and automated quantification of shunts and valve lesions, with direct relevance to pediatric structural and hemodynamic interventions [3,16]. Their article highlights AI-enabled 3D modeling, image fusion, and virtual planning tools that support pediatric interventional cardiology procedures, as well as early experiences with AI for risk prediction, long-term outcome modeling, and remote monitoring.

The regulation of AI software as a medical device is evolving. The ACR Pediatric AI Workgroup calls for public information on training data, age-stratified performance, and pediatric evidence, ideally as an “AI nutrition label” [3,10]. A multisociety pediatric radiology statement addressed regulation, purchasing, implementation, integration, interpretation, post-market surveillance, and education, proposing pediatric-specific safety ratings, transparency metrics, and explainability requirements [10,13]. From a PIR perspective, these remain diagnostic-centric and seldom address procedural, anesthetic, and perioperative concerns.

From a PIR perspective, these AI methods are clinically relevant not as standalone computational techniques, but as enabling tools for dose-sparing image enhancement, automated segmentation, device and access planning, procedural navigation, workflow optimization, and postprocedural risk prediction [3,8,16].

### 2.2. Challenges in Implementing AI in Pediatric Patients

The implementation of AI in pediatric patients is limited by technical, ethical, regulatory, and economic constraints. Imaging protocols emphasize radiation reduction, yielding lower signal-to-noise ratios and altered contrast, and magnetic resonance (MR) sequences are frequently shortened to reduce or avoid sedation [10,11,12,13,14,15,16,17,18,19]. Algorithms trained on adult high-dose CT or prolonged MR often underperform on pediatric images, especially in young children [3,10,20]. DL-reconstructed low-dose CT is now commonly used for diagnostic pediatric imaging; however, vendor-neutral tools for fluoroscopy and cone-beam CT are scarce, forcing trade-offs between dose and intraprocedural confidence [3,10,20].

Children vary widely in size, anatomy, and physiology, from premature neonates to bariatric adolescents [11,15], and age-dependent changes in the thymus, marrow, perfusion, and biometrics influence appearance and disease patterns [11,15]. Thus, training datasets must represent this heterogeneity to avoid biased performance in underrepresented ages, sizes, or pathologies; even “large” multicenter PIR cohorts become small once stratified by age, weight, and disease [10,11,15]. Indeed, data scarcity is a central challenge in implementing AI in pediatric patients. Public datasets are predominantly based on adults, and the time for expert labeling by pediatric radiologists is limited [6,10,15]. Children are a protected population; thus, research must navigate stricter consent, assent, and privacy rules, including secondary use, data ownership, and the right to withdraw in adulthood [6,10,15]. Low procedure volumes and reimbursement further weaken the commercial incentives for researching pediatric-specific AI [6,10,15]. Moreover, ethical issues include algorithmic bias, “black box” opacity, unclear liability, and the risk that adult-trained AI could disadvantage children—for example, via AI-triaged worklists [10,13,14]. In line with this, Sammer et al. reported that adult algorithms for vertebral fractures, chest radiographs, and thyroid nodules performed substantially worse in children than in adults, especially those younger than 2 years of age or those with cardiomegaly, emphasizing the need for pediatric-specific validation [10]. Similar cautions appear in broader pediatric AI and neuro-oncology, where AI response assessment in pediatric neuro-oncology frameworks addresses childhood central nervous system tumor biology and imaging [10,13,14].

Pediatric imaging often depends on public or lower-reimbursement insurance schemes (such as Medicaid in the United States), limiting investment in AI infrastructure, licensing, and monitoring; hence, most tools are treated as practice expenses rather than reimbursed services [10,11,15]. For PIR services, which are already constrained in equipment and staffing, additional AI investment requires clear gains in safety, throughput, or reimbursement, metrics that are rarely reported in current AI studies [10,11,15].

Collectively, these issues—limited pediatric data and labeling resources, ethical and legal concerns around algorithmic bias and opacity, and the predominance of adult-trained models—form a common constraint that recurs across PIR-relevant AI applications. Rather than being repeated in each subsection, these considerations should be considered as a common backdrop: most of the tools and examples discussed in this review inherit the same risks of under-representation, uncertain generalizability to small or atypical children, and the need for dedicated pediatric validation before routine use.

### 2.3. Applications of AI in PIR

#### 2.3.1. Image Acquisition and Reconstruction

Radiation reduction offers a strong opportunity for the use of AI in PIR. In interventional cardiology and related fluoroscopy, eye tracking and ML have shown that a substantial proportion of fluoroscopy time (>11 min per procedure) occurs while the operator is not actively viewing the monitor, representing avoidable radiation exposure [10,21]. AI-equipped fluoroscopy systems with ultrafast region-of-interest collimation substantially reduce the patient dose-area product and staff scatter compared with conventional systems, even after adjusting for patient characteristics and fluoroscopy time [17,18]. Although studied in adult endoscopy, the principle of dynamic collimation guided by real-time image analysis and operator behavior is highly relevant to pediatric fluoroscopy and angiography [10,21,22].

DL-based CT and MR reconstruction improves image quality at a lower dose or shorter acquisition time by learning noise and artifact removal from paired low- and standard-dose data. In 80-kVp pediatric CT, DL reconstruction has been shown to enable a dose reduction of approximately 50% while preserving or improving image quality metrics, including noise levels, signal-to-noise ratio, and contrast-to-noise ratio, compared with standard iterative methods. Moreover, radiologists preferred DL images [11]. These findings support lower-dose pediatric volumetric imaging and suggest an extension to digital subtraction angiography and cone-beam CT, where DL could denoise low-pulse-rate fluoroscopy, enhance vessel contrast from sparse projections, or synthesize higher-quality volumes from fewer rotations, potentially shortening the duration of anesthesia and reducing contrast [11,20]. Pediatric interventionalists rely on CT for access mapping, device sizing, and postprocedural assessment. Thus, achieving these at lower doses without compromising confidence is directly relevant to PIR, even if CT sits “outside” the angiography suite [3,5,20].

ML systems trained on historical data can link patient factors (age, weight, and habitus) and indications to protocol parameters (kV, mA, frame rate, and filtration) that balance image quality and dose [10,11]. Reinforcement learning agents can refine such protocols in simulations before cautious clinical deployment [11]. AI-assisted protocol selection integrated into interventional consoles could provide patient- and indication-specific presets aligned with the “Image Gently” principles [10,11].

#### 2.3.2. Image Interpretation and Diagnosis

Although PIR is procedure-focused, interpretation underpins case selection, route planning, and intraprocedural decisions. In pediatric cardiology, DL achieves near-expert performance in arrhythmia detection using single-lead electrocardiograms (ECGs), automated view classification, chamber quantification, myocardial strain analysis, and MRI-based prognostic modeling, demonstrating that high-performing AI is feasible when large pediatric datasets are available [4,16,19]. Most PIR image interpretations, such as assessing venous collaterals, access feasibility, or small infarcts, remain manual.

DL-based segmentation of organs, vessels, and lesions on CT, MRI, and ultrasonography enables volumetry, distance measurement, and risk assessment. Automated hepatic segmentation can streamline portal vein recanalization, transjugular intrahepatic portosystemic shunts, ablation, and embolization planning in children with portal hypertension, tumors, or vascular anomalies. Venous segmentation also supports stent planning for central venous occlusion or malformations, both of which are major indications for PIR [3,5]. CNN-driven segmentation powers many procedural toolkits, underpinning centerline extraction, lesion volumetry, and path-length estimation for device selection [3,5]. However, most currently available segmentation algorithms and procedural toolkits have been trained and validated using adult datasets, and their performance in small or anatomically variant pediatric patients remain incompletely characterized, highlighting the need for pediatric-specific training and validation before routine PIR deployment [8].

Predictive models combining imaging and clinical data can support diagnostic classification or outcome estimation. For example, dynamic Bayesian networks and dynamic network analysis have identified inflammatory mediators associated with spontaneous recovery versus transplantation in cases of pediatric acute liver failure [5]. In children awaiting liver transplantation, random forest models using clinical and trajectory variables improved wait-list outcome prediction [5]. If integrated into PIR pathways (e.g., portal hypertension, biliary complications, and vascular malformations), such models could identify children most likely to benefit from early intervention versus observation, potentially avoiding high-risk procedures [3,4,5,6]. Moreover, Holt et al. illustrated task-specific pediatric AI by integrating echocardiographic and clinical data for the automated detection and hemodynamic assessment of atrial septal defects [16]. Ayaz et al. reported that CNNs can detect gastrointestinal obstruction on pediatric radiographs with high diagnostic accuracy, potentially supporting AI-assisted triage and pre-interventional assessment in emergencies [23]. These systems support clinicians rather than replace them and can considerably influence PIR case selection and strategy. Furthermore, Sammer et al. reported that adult-trained AI often fails to generalize to children, with underperformance in vertebral fracture, chest radiography, and thyroid nodule algorithms [10]. For PIR, this underscores the need for pediatric-specific training and validation for AI-influencing indications, access, or device choice [11,13].

#### 2.3.3. Procedural Planning and Guidance

PIR planning is increasingly involving 3D/four-dimensional imaging, 3D printing, procedural software, and mixed reality. AI-assisted modeling can segment vessels and organs using CT/MR or cone-beam CT to create patient-specific models for virtual rehearsal, device sizing, and access assessment [5,8,11]. In pediatric transplantation, ML models integrating clinical and pharmacogenomic data have predicted tacrolimus kinetics and graft outcomes, suggesting that analogous PIR models could support individualized selection of shunt diameters, stent sizes, and embolic volumes [5,8,11].

Augmented reality (AR) concepts, such as Philips–Microsoft HoloLens, project live imaging and vital signs into a manipulable 3D workspace, maintaining sterility through voice or gestures [3]. Combined with AI-derived segmentation and catheter-path prediction, AR can overlay targets during pediatric interventions, thereby shortening procedures and reducing contrast [8,19]. DL-based hand gesture recognition allows accurate, low-latency, touchless control of viewers or robotic interfaces [8,19].

In preclinical settings, deep reinforcement learning has trained robotic manipulators to navigate guidewires through complex vascular phantoms with high success rates, illustrating its potential for semi-autonomous navigation [3]. Pediatric-scaled adaptations can support complex congenital neurovascular or portal venous interventions [3,8,24]. Procedural toolkits integrating image fusion, vessel tracking, device libraries, and quantitative planning have been established for adults and are increasingly being applied in PIR. In practice, their main value lies in faster reproducible measurements, standardized segmentation, and fewer on-table surprises [3,8]. As AI components such as automated centerline extraction, path planning, and real-time registration are added, these toolkits may further enhance the software’s usefulness in PIR [8,24].

A smart speaker application using natural language processing has been shown to answer real-time questions regarding device compatibility and sizing across hundreds of IR devices with excellent performance, suggesting a path toward cloud-based, on-demand procedural reference tools [15,25]. In pediatrics, where devices are often adapted from adult inventories and sizing margins are narrow, such tools may reduce delays and errors [3,15,25]. Moreover, MRI-guided cardiac catheterization in children has integrated DL-based balloon and catheter tracking with real-time imaging to maintain slice alignment and improve device conspicuity. This demonstrated tightly coupled AI–imaging–navigation loops that align with radiation-free guidance needs in complex PIR [13,16].

#### 2.3.4. Post-Procedure Monitoring and Follow-Up

Post-procedural PIR monitoring is well-suited to AI-based risk prediction and anomaly detection. In pediatric liver transplantation, ML models have predicted wait-list outcomes, early graft failure, and rejection from perioperative variables, laboratory trajectories, and transcriptomic profiles, providing high-discrimination risk stratifications [5]. For PIR, such probabilities can better structure discussions about surveillance and discharge than an informal gestalt.

In IR, ML prediction of responses to intra-arterial hepatocellular carcinoma therapies offers a template for embolization and ablation outcome models [3,5]. However, these outcome-prediction frameworks were developed in predominantly adult populations, and their structure is currently used as a conceptual template rather than an already validated model for PIR [3,5,9]. Similar frameworks in PIR could estimate re-bleeding risk after hemorrhage control, complications after vascular tumor embolization, or stenosis after venous stenting, guiding tailored surveillance and early re-intervention. Advanced models can integrate procedural parameters, immediate post-procedural imaging, and early laboratory markers [3,5,9]. At present, such PIR-specific models remain largely hypothetical or at an early exploratory stage; critical gaps include prospective multicenter validation, age- and diagnosis-stratified performance reporting, and demonstration of impact on surveillance strategies and clinical outcomes [3,5,15].

Automated systems can analyze follow-up ultrasonography, CT, or MRI for stent thrombosis, bile leak, abscess, or malformation progression, flagging studies for priority review, while AI-based monitoring of electronic records, similar to early warning systems in pediatric intensive care, can detect subtle deterioration after complex PIR [3,6,12]. Most such systems are adapted from diagnostic radiology or pediatric intensive care settings and have not been systematically evaluated in PIR workflows; their sensitivity, specificity, and false-alarm burden in the immediate post-interventional period remain key areas for future validation [3,6,12]. These tools should augment rather than replace clinical vigilance measures. Parental and nursing observations often precede quantified deterioration, and PIR-specific models must consider this [3,6,12]. Desai et al. and others have suggested that predictive tools can be employed to support anticipatory resource allocation, such as enhanced monitoring for high-risk children [3,6,12]. Across these applications, an important limitation is that many commercially available or prototype systems were developed using adult or mixed-age data; therefore, their performance and safety in children must be regarded as extrapolations until age-stratified validation and PIR-specific outcome data are available.

## 3. Future Directions

### 3.1. Emerging AI Technologies

Although DL is well established in many areas of medical imaging, it is still emerging in pediatric interventional radiology, as advanced DL architectures such as transformers and foundation models have not yet been applied to PIR data in a systematic way. Explainable artificial intelligence (XAI) refers to methods that make ML predictions interpretable to humans, for example, through visualizations, feature importance rankings, or local surrogate models [4,5,14]. In pediatric transplantation and cardiology, interpretable models have predicted outcomes after congenital heart surgery and transplantation, while clarifying which variables drive predictions and increasing clinician confidence [4,5,14]. For PIR, XAI may be essential to overcome discomfort with opaque recommendations. Federated learning is a distributed training approach in which centers jointly train models by sharing model parameters or gradients rather than raw patient data, thereby preserving local data privacy [6]. This is desirable for rare PIR indications in which single-center datasets are underpowered. Pediatric transplantation, perinatology, and neuro-oncology networks have demonstrated harmonized data dictionaries and governance for multi-institutional AI research, providing a PIR template [5,6,14]. Multimodal integration, which combines imaging, clinical variables, laboratory data, genomics, and wearables, is an important frontier for future research. In pediatric cardiology, AI-enabled phenotype profiling aims to refine disease subtypes and guide precision medicine. Similar work in perinatology and neuro-oncology integrates multiomics and AI for risk stratification and individualized therapy [4,6,14]. To date, these models have been evaluated primarily in retrospective or single-center cohorts and remain research tools rather than PIR-integrated decision support systems, with prospective validation and external benchmarking still needed [5]. For PIR, analogous models could inform choices between interventional, surgical, or medical management for portal hypertension, complex vascular malformations, and transplant-related complications [4,6,14]. At present, most literature on XAI, federated learning, and multimodal integration in pediatrics is related to cardiology, transplantation, perinatology, or neuro-oncology, with little direct PIR-specific validation; in PIR, these concepts should therefore be regarded as promising research directions rather than established clinical tools [4,5,6,14]. These models often assume disease phenotypes, imaging protocols, and outcome measures that differ from interventional workflows; thus, their relevance to PIR is best viewed as conceptual—highlighting feasible architectures and collaboration models—rather than ready-made solutions for interventional practice [4,5,6,14].

Clinician trust in AI-derived segmentations, navigation suggestions, and risk predictions will depend on transparent performance reporting (including age-stratified accuracy and calibration), clear indication of uncertainty, and, where feasible, explainable interfaces that reveal the main factors driving a recommendation. In addition, governance frameworks should define how AI-supported decisions are documented and audited, and how responsibility is shared when AI outputs contribute to complications, so that teams can incorporate these tools with confidence rather than concern.

### 3.2. Potential Impact on Clinical Practice

AI can streamline workflows, improve diagnostic and procedural accuracy, and support personalized PIR care if carefully governed. Early pilot experience suggests that AI-assisted scheduling can meaningfully shorten PIR outpatient waiting times and improve clinic throughput; nonetheless, these reports are based on small, single-center cohorts without controlled comparison and should therefore be interpreted with caution [3,7,15]. Smart assistants using natural language processing may similarly help triage calls, answer common questions, and retrieve device information, potentially reducing cognitive burden and mitigating burnout, although these applications also remain at an early developmental stage [3,15,26].

Automated segmentation and quantitative analysis can be used to standardize planning measurements, whereas outcome-prediction models can provide reproducible risk estimates. This is particularly valuable when a small number of pediatric interventionalists cover large regions [5,11]. For smaller centers, AI-enabled decision support embedded in planning software can offer a virtual subspecialty backup if trained and validated responsibly [3].

Personalized treatment planning is another promising approach. Small-data AI platforms in pediatric transplantation have supported individualized tacrolimus dosing predictions, suggesting the feasibility of personalized control of embolic volumes, ablation energy, or stent dimensions based on anatomy, hemodynamics, and prior responses to PIR [3,5,8]. To this end, AI-enhanced procedural software may increasingly act as a central hub aggregating imaging, planning, navigation, and post-procedural analytics for each child [8]. When extrapolated from experience in pediatric cardiology, transplantation, perinatology, or neuro-oncology, these examples are intended as hypothesis-generating concepts for PIR rather than as evidence of fully validated interventional tools and should therefore be interpreted as forward-looking clinical opportunities [4,5,6,14].

In practice, AI and robotic functions are most likely to be adopted when they are embedded into the procedural software, angiography consoles, and planning tools that PIR teams already use, rather than as stand-alone applications. Integration should align AI outputs with specific decision points along the PIR workflow; for example, automatic segmentations and centerlines embedded within planning viewers, dose-optimization presets integrated into the protocol-selection interface, and risk scores displayed in peri-procedural dashboards to enhance existing workflows without disrupting clinical practice.

### 3.3. AI-Adjacent Robotic and Smart Navigation Systems

Robotic and smart navigation systems are reshaping endovascular practice and providing a technological basis for future PIR, although none have yet been designed or validated specifically for children [26,27,28,29]. The LIBERTY endovascular robotic system (Microbot Medical) is a single-use, remotely operated platform for peripheral interventions, enabling tele-operated guidewire and catheter manipulation from a shielded workstation. Early reports and company communications emphasized potential reductions in operator radiation and musculoskeletal strain, and the system has FDA 510(k) clearance with pivotal evaluations in progress [26,27,28,29]. Reusable platforms, such as CorPath GRX and Magellan, have demonstrated high technical success and reduced operator doses in coronary, carotid, and peripheral interventions, and reviews have highlighted their ability to integrate high-fidelity imaging, geometric modeling, and machine-vision assistance as a basis for AI-driven path planning and semi-autonomous navigation [28,29,30,31] (Table 1).

**Figure 1:**
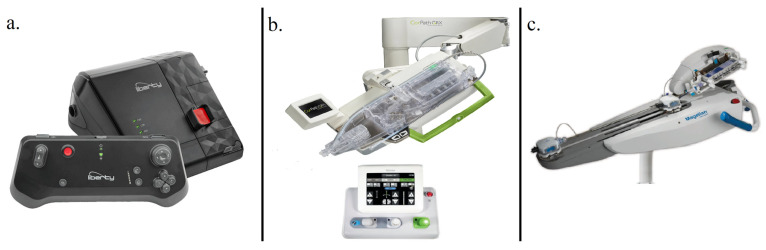
Examples of artificial intelligence-adjacent robotic and smart navigation systems intended for interventional radiology. The illustration emphasizes generic technological capabilities—such as remote wire and catheter manipulation from a shielded workstation, high-fidelity kinematic and procedural data capture, and integration with advanced imaging and geometric modeling—which together provide a basis for future machine-learning–assisted path planning, collision avoidance, and semi-autonomous navigation, particularly in complex vascular anatomies. Specific commercial systems are shown to exemplify these concepts and are included for educational illustration only; their depiction does not imply endorsement, preference, or comparative evaluation, and the author has no financial relationship with the manufacturers. (**a**) LIBERTY by Microbot Medical (https://microbotmedical.com/liberty/; accessed on 18 June 2026). (**b**) CorPath GRX by Corindus, a Siemens Healthineers Company (https://www.dicardiology.com/content/corindus-seeking-neurovascular-intervention-clearance-corpath-grx-vascular-robotic-system; accessed on 18 June 2026). (**c**) Magellan Robotic System by Hansen Medical (now Auris Health; https://vascularnews.com/auris-surgical-robotics-agrees-to-acquire-hansen-medical/; accessed on 18 June 2026).

Currently, LIBERTY, CorPath GRX, RobEnt, and Magellan are regarded as AI-adjacent hardware. Control remains rule-based and operator-directed, although it generates rich procedural, kinematic, and force data streams that could support future ML-based path planning, collision avoidance, dose optimization, and semi-autonomous navigation once curated and modeled [26,27,28,29,30,31,32] (Figure 1). From a pediatric perspective, they illustrated how robotics and remote navigation could support safer catheterization in small tortuous vessels and even centralized expert intervention with local teams; however, no system has yet been optimized, labeled, or systematically evaluated for PIR [3,26,33].

Beyond their technical promise, these robotic platforms are constrained by substantial capital and maintenance costs, dedicated space and shielding requirements, and the need for compatible imaging infrastructure, which currently limits their deployment to a small number of well-resourced centers. For many pediatric institutions, especially those with lower procedural volumes, such costs and logistical demands may outweigh potential benefits unless clear gains in operator safety, procedural efficiency, and patient outcomes can be demonstrated and supported by appropriate reimbursement models.

### 3.4. AI-Enabled Handheld Robotic Ultrasound Guidance for Vascular and Organ Access

**Figure 2:**
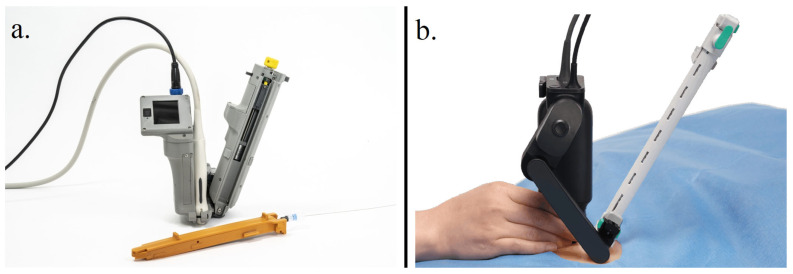
Examples of artificial intelligence (AI)-enabled handheld robotic ultrasound guidance for vascular and organ access intended for interventional radiology. (**a**) Artificial Intelligence Guided Ultrasound Interventional Device (AI-GUIDE), developed to enable non-expert users to perform ultrasound-guided catheterization of deep vessels such as the femoral vein. (**b**) Handheld robotic ultrasound-guided needle intervention system by Mendaera Inc., cleared by the United States Food and Drug Administration (510(k)) to assist with needle-based procedures and to reduce user variability and enhance procedural efficiency.

AI-enabled ultrasound-guided handheld or compact robotic devices aim to deliver rapid and accurate vascular or organ access at the patient’s bedside, which is especially relevant for unstable children when PIR expertise is not immediately available [34,35,36,37]. The AI-GUIDE platform clips onto a standard linear probe, uses onboard AI to detect the femoral vein on live ultrasonography, and drives an integrated needle until venous entry, enabling non-experts in preclinical models to perform central access with near-expert performance and suggesting a future role in pediatric emergencies once miniaturized and validated [34]. HUMaN combines a handheld ultrasound device with magnetic needle tracking to display the true needle position and predicted trajectory, thereby reducing the error associated with steep or poorly visualized paths and offering a template for pediatric deep venous or organ access [34]. Compact, cooperatively controlled robotic probe holders share control of the probe position and contact force, enabling stable, repeatable probe placement and tissue deformation for radiotherapy monitoring, and offering a hardware paradigm that AI could extend to motion-compensated, standardized ultrasound guidance and longitudinal PIR follow-up [34,35,36,37]. The Mendaera Focalist handheld robotic system recently received FDA 510(k) clearance for ultrasound-guided needle procedures in adult and pediatric patients, representing the first regulator-recognized commercial implementation and underscoring the clinical need for PIR-specific evaluation [37] (Table 2 and Figure 2). Contrarily, AI-GUIDE, HUMaN, and cooperative ultrasound robots remain preclinical or prototype systems without prospective pediatric clinical studies; therefore, their proposed roles in PIR should currently be viewed as investigational and hypothesis-generating [34,35,36]. Similarly, for more compact handheld systems, broad adoption will depend on practical factors such as device affordability, integration with existing ultrasound platforms, and availability of technical support across diverse practice settings. In addition, safe use requires structured training in both ultrasound and robotic workflows, clear delineation of responsibilities between interventionalists, anesthesiologists, and bedside clinicians, and robust guidance on credentialing and ongoing competency assessment.

The illustration focuses on core technological principles, including AI-assisted vessel detection on real-time ultrasound, robotically stabilized or guided needle trajectories, and feedback mechanisms designed to improve the accuracy, safety, and reproducibility of needle-based procedures, particularly for users with limited ultrasound or interventional experience. Specific systems are depicted to exemplify these concepts and are provided for educational illustration only; their inclusion does not imply endorsement, preference, or comparative evaluation, and the author has no financial relationship with the manufacturers.

(a) Artificial Intelligence Guided Ultrasound Interventional Device (AI-GUIDE), developed to enable non-expert users to perform ultrasound-guided catheterization of deep vessels such as the femoral vein.
(b) Handheld robotic ultrasound-guided needle intervention system by Mendaera Inc., cleared by the United States Food and Drug Administration (510(k)) to assist with needle-based procedures and to reduce user variability and enhance procedural efficiency.

### 3.5. Research Priorities and Collaborative Studies

**Figure 3:**
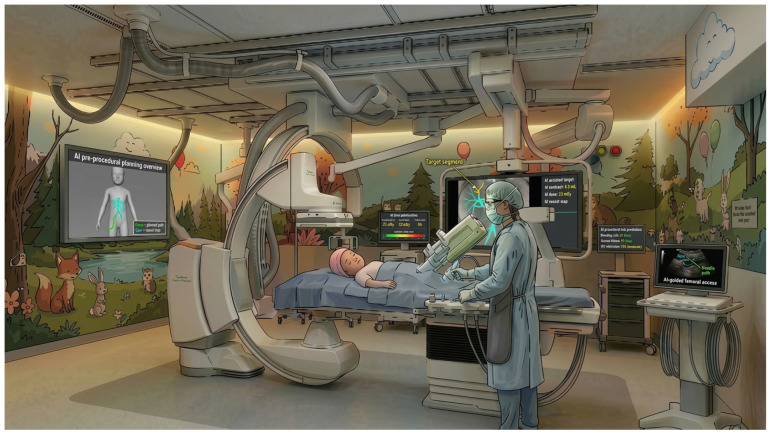
Fictional artificial intelligence (AI)-enabled pediatric interventional radiology suite. Concept illustration of a near-future angiography suite, created by the author, in which multiple AI tools support the pediatric interventional radiology workflow from planning to access. The left-wall screen depicts AI-assisted pre-procedural planning with three-dimensional vascular modelling and an optimized catheter path; the central fluoroscopy display shows AI fusion with vessel segmentation and a highlighted target branch to facilitate real-time targeting and tracking. Two small side monitors present AI-driven radiation-dose monitoring and procedural risk prediction, while AI-enabled and robotic-ready devices adjacent to the table provide intraprocedural navigation support. The right ultrasound screen illustrates an AI-guided vascular access planner for femoral venous puncture. This figure is a schematic, non-commercial integration of AI components already reported individually in the literature and is intended as a didactic, hypothesis-generating depiction of a potential future PIR environment rather than as a representation of any specific existing clinical suite.

The priorities for PIR-relevant AI can be summarized as follows: (i) large, harmonized pediatric datasets with detailed procedural and outcome annotations; (ii) externally validated procedure-specific predictive models; (iii) integration of AI components into existing procedural software (Figure 3); and (iv) socio-technical studies on usability, trust, and training [3,8,15]. In addition to usability and trust, structured training is essential: PIR attendings, fellows, technologists, and nurses will need to undergo curricula covering basic AI concepts, system limitations and failure modes, and hands-on experience with AI-enabled consoles and robotic interfaces. Simulation-based training and supervised proctoring for new systems, together with clear institutional policies on credentialing and ongoing competency assessment, will be critical to ensure safe deployment. A recent needs-assessment survey of pediatric interventional radiologists found active research participation but highlighted a lack of support staff, fragmented infrastructure, and limited research training as key barriers, alongside a strong interest in collaborative research on interventional oncology, vascular anomalies, venous thrombosis, and venous access, all of which are well-suited to AI-enabled quantitative imaging and outcome modeling [3,15]. These research themes should be interpreted as hypothesis-generating priorities for PIR, extrapolated from current experience in pediatric cardiology, transplantation, perinatology, and neuro-oncology rather than from mature, PIR-specific evidence, and are therefore presented as an aspirational framework to guide future endpoint-driven studies [3,4,5,6,14,15].

Looking ahead, AI research in PIR should explicitly focus on pediatric clinical endpoints that matter in day-to-day care—such as cumulative effective dose, fluoroscopy time, contrast volume, and how long children need sedation or anesthesia—alongside procedural success, 30-day complication and re-intervention rates, length of stay, and patient-reported outcomes, so that new tools are judged by their real impact on children and their families. Dedicated PIR collaboratives with central coordination, standardized protocols, and shared data/statistical infrastructure are key strategies for overcoming these barriers. Aligning PIR collaboratives with broader pediatric AI initiatives from radiology and pediatric societies could ensure that PIR needs are represented in funding, regulatory, and reimbursement frameworks. Prospective multicenter comparisons of AI-augmented versus standard workflows will be essential to demonstrate real-world benefits and guide implementation. In this context, the pediatric AI gap described by the ACR Pediatric AI Workgroup [10]—where only a small fraction of cleared AI tools are explicitly useful in children and most are trained predominantly using adult datasets—underscores our conclusion that AI for PIR remains technically feasible but rarely designed or validated specifically for pediatric endpoints [3,10,11,15].

As a narrative, clinically oriented review, this article summarizes illustrative examples from heterogeneous AI and robotic studies without applying a formal, tool-based risk-of-bias assessment. Many cited reports are small, single-center or retrospective series, and effect sizes are quantified only in selected instances where robust pediatric data are available, such as low-dose CT reconstruction, while other examples remain primarily descriptive [10,13,14,15]. Positive findings are therefore not systematically contrasted with neutral or negative results, and extrapolations from adult IR, cardiology, and transplantation introduce an inherent citation and selection bias. Future PIR-focused AI research should not only explore whether proposed tools improve diagnostic, procedural, or workflow metrics, but also report standardized quantitative measures (including effect sizes and uncertainty) and incorporate basic bias assessment, so that benefits, limitations, and failures can be compared and synthesized across centers in a more rigorous manner [10,13,14,15].

Overall, the successful integration of advanced AI and robotic systems into PIR will require not only technical maturity but careful consideration of cost-effectiveness, equity of access between high- and low-resource centers, and the significant training and change-management efforts needed to embed these tools safely into daily practice. Without explicit attention to these factors, such technologies risk widening existing disparities in pediatric interventional care rather than narrowing them.

## 4. Conclusions

AI has the potential to improve care but has not yet been designed, validated, or deployed specifically for children. Existing work demonstrates its feasibility across the PIR workflow—dose-sparing fluoroscopy and DL reconstruction, automated segmentation and planning, AI-assisted navigation and robotics, and workflow optimization and outcome prediction—but most tools remain weakly linked to pediatric clinical endpoints. Meaningful progress depends on rigorously designed, pediatric-specific studies that demonstrate improvements in radiation exposure, procedural efficiency, safety, and patient-centered outcomes. If pediatric interventional radiologists work in close partnership with diagnostic pediatric radiologists, data scientists, engineers, ethicists, regulators, payers, and patient advocates, AI can move from a peripheral add-on to a trusted, accountable layer within the PIR infrastructure, thereby improving precision, safety, and equity in children who require image-guided interventions.

## Figures and Tables

**Table 1:** Documented features of contemporary endovascular robotic systems and their AI-adjacent elements (representative selection of widely reported platforms; not exhaustive).

System	FDA Cleared	Use Type	Operator Position	Evidence Setting	Cost (Reported)	Key Technical Feature	Pediatric Use *	AI Contribution
LIBERTY [27,28]	Peripheral indication noted in the transarterial review	Single-use endovascular module	Remote, shielded workstation	Porcine model; early peripheral use	“Lower capital barrier” concept only	Table-mounted, single-use, wire/catheter control	None reported	Hardware platform for future AI navigation
CorPath GRX [29,30]	PCI and PVI indications summarized	Reusable console, disposable cassette	Remote cockpit outside the field	PCI/PVI; aneurysm embolization	High-cost capital system	Robotic wire/catheter control; automation functions	None reported	Data source for ML-based path planning
Magellan [31,32]	Peripheral robotic catheter clearance discussed	Reusable platform, single-use catheters	Remote console	Peripheral uterine chemoembolization	High initial and disposable costs	6–10-French multi-bend catheters	None reported	Candidate platform for AI-assisted navigation

* No indexed reports to date document pediatric-specific indications, labeling, or clinical series for LIBERTY, CorPath GRX, or Magellan; any pediatric use would therefore be off-label and extrapolated from adult IR practice or experimental experience. Abbreviations: AI—Artificial Intelligence; FDA—Food and Drug Administration; PCI—percutaneous coronary intervention; PVI—peripheral vascular intervention.

**Table 2:** Key characteristics of AI-enabled handheld or compact ultrasound–robotic systems relevant to pediatric interventional radiology.

System *	Intended Task	Control Paradigm	US–Needle Relationship	Evidence Setting	AI Contribution
AI-GUIDE-type device [34]	Automated femoral venous access (porcine)	Operator holds probe; robot drives needle	Clips onto the linear probe; AI localizes the vein	Preclinical porcine central access study	CNN-based vein detection and trajectory planning
HUMaN system [35]	Real-time 3D needle guidance	Operator advances needle; system guides visually	Handheld US with magnetic needle tracking overlay	Phantom/preclinical targeting evaluations	EM tracking and real-time registration
Cooperative US robot [36]	Stable probe for RT monitoring	Shared human–robot probe control	Robot holds probe; no needle integration	Prototype during radiotherapy monitoring	Force/position sensing; cooperative control algorithm
Mendaera Focalist system [37]	Ultrasound-guided needle placement (multiple procedures)	Handheld robot aligns, positions, and tracks the needle	Attaches to probe; guides instrument relative to US	FDA 510(k)-cleared; limited clinical launch	Advanced software for targeting and depth tracking

* All but one of these platforms remain unpriced, non-cleared prototypes or research systems without prospective pediatric evaluation; only the Mendaera handheld robotic system is FDA-cleared with labeling that includes pediatric use, and even for this device, PIR-specific performance, workflows, and outcomes will require dedicated study and validation. Abbreviations: CNN, convolutional neural network; EM, electromagnetic; FDA, Food and Drug Administration; RT, radiation therapy; 3D, three-dimensional; US, ultrasound.

## Data Availability

The data are available from the corresponding author on reasonable request (amsharydah@iau.edu.sa).

## Abbreviations

The following abbreviations are used in this manuscript:

ACR	American College of Radiology
AI	Artificial Intelligence
AI-GUIDE	Artificial Intelligence–Guided Ultrasound Intervention Device
AR	Augmented Reality
CAD	Computer-Aided Diagnosis
CNN	Convolutional Neural Network
CT	Computed Tomography
DL	Deep Learning
DLR	Deep Learning–Based Reconstruction
ECG	Electrocardiogram
FDA	Food and Drug Administration
HUMaN	Handheld Ultrasound System with Magnetic Needle Navigation
IR	Interventional Radiology
ML	Machine Learning
MR	Magnetic Resonance
MRI	Magnetic Resonance Imaging
PIR	Pediatric Interventional Radiology
XAI	Explainable Artificial Intelligence
